# Lipid nanoparticles: Composition, formulation, and application

**DOI:** 10.1016/j.omtm.2025.101463

**Published:** 2025-04-08

**Authors:** Sijia Xu, Zhenzhen Hu, Fenglin Song, Ying Xu, Xuexiang Han

**Affiliations:** 1 Key Laboratory of RNA Innovation, Science and Engineering, CAS Center for Excellence in Molecular Cell Science, Shanghai Institute of Biochemistry and Cell Biology, Chinese Academy of Sciences, University of Chinese Academy of Sciences, Shanghai 200031, China; 2 School of Pharmacy, East China Normal University, Shanghai 200241, China

**Keywords:** lipid nanoparticles, nucleic acid, composition, formulation technique, therapeutic applications

## Abstract

Lipid nanoparticles (LNPs) are lead non-viral vectors for delivering nucleic acids. LNPs can efficiently encapsulate nucleic acids, protect them from degradation, enhance cellular uptake and induce endosome escape, which show high transfection efficiency and low immunogenicity. In this review, we first introduce the LNP components, highlighting their critical roles in encapsulation, stability, delivery efficiency, and tissue tropism. Next, we summarize different techniques for LNP formulation with a focus on their advantages and disadvantages. Then, we discuss the diverse applications of LNPs in preclinical and clinical studies. Finally, we provide perspectives in the future development of LNPs.

## Graphical abstract

**Figure:**
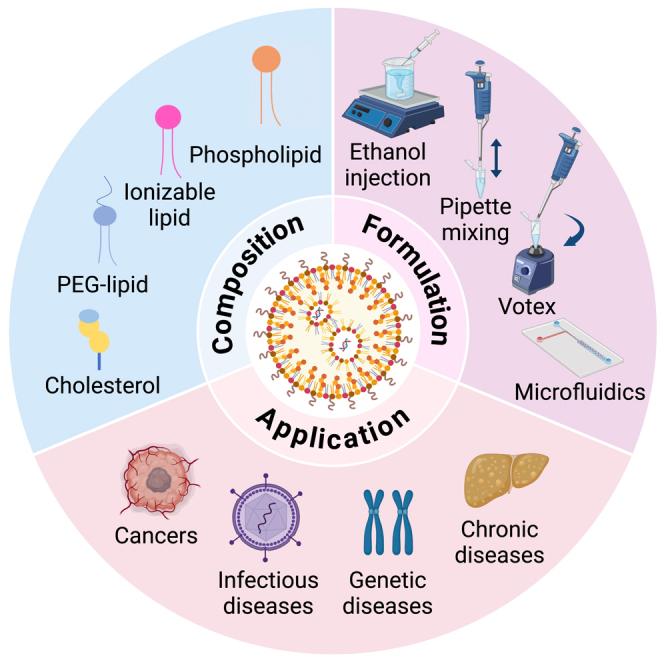

## Teaser

In this review, Han and colleagues systematically discuss the composition, formulation techniques, and application of lipid nanoparticles.

## Introduction

Nucleic acids have emerged as important therapeutic agents, with broad biological and clinical applications. However, the delivery of nucleic acids remains challenging due to their instability, negative charge, and large size, necessitating additional vectors to effectively penetrate cell membranes.^1^^,^^2^ Viral vectors are widely used for gene delivery, but have some disadvantages, including high immunogenicity, limited package capacity, and potential carcinogenesis.^3^^,^^4^

The aforementioned disadvantages of viral vectors can be overcome by lipid nanoparticles (LNPs). LNPs have been harnessed to deliver different types of nucleic acids, such as small interfering RNA (siRNA), messenger RNA (mRNA), and plasmid DNA.^2^ These encapsulated nucleic acids can be protected from degradation, and be released into cytoplasm after endosome escape to achieve the desired purposes.^5^ Currently, LNPs are broadly utilized in the development of RNA medicine and have contributed a lot in disease prevention and treatment. For example, Pfizer-BioNTech used ALC-0315 LNP to successfully deliver mRNA vaccine against the SARS-CoV-2 virus.^6^ Besides, LNPs are widely used in immunotherapy, gene editing, protein replacement therapy, cancer vaccine, and so on.^7^^,^^8^^,^^9^ Many efforts have been put into the optimization of LNP components, such as optimizing the molar ratio and the chemical structure of ionizable lipid, which could greatly influence the tissue tropism, delivery efficiency, and safety of LNPs.^10^^,^^11^ Besides, formulation techniques critically affect LNP stability, scalability, and batch-to-batch variability.^12^

In this review, we first introduce the LNP components, highlighting their critical roles in encapsulation, stability, and delivery efficiency. Next, we summarize different methods for LNP formulation with a focus on their advantages and disadvantages. Finally, we discuss the applications of LNPs in the treatment of cancers, infectious diseases, genetic disorders, and other pathological conditions.

## Composition

**Figure 1:**
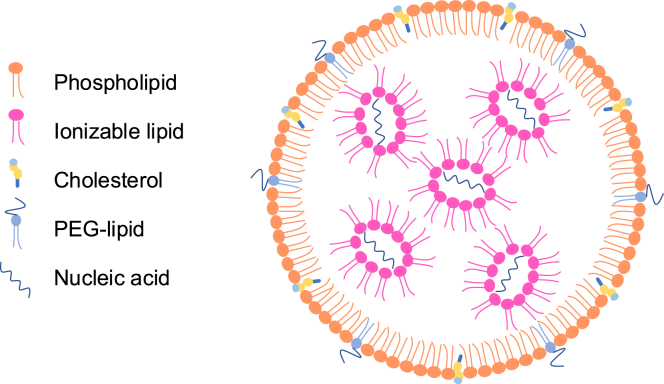
Typical composition of LNPs Conventional LNPs have four components, including ionizable lipids, PEG-lipids, phospholipids, and cholesterol, which are used to deliver nucleic acids, especially RNA molecules.

LNPs typically comprise four components (Figure 1): (1) ionizable lipids, which play the most important role in encapsulating and delivering nucleic acids; (2) polyethylene glycol (PEG)-lipids, which determine the size and stability of LNPs; (3) phospholipids, which affect the fusogenicity of LNPs; and (4) cholesterol, which improves the stability and fluidity of LNPs. Apart from conventional four-component LNPs, some LNPs contain additional components to improve the targeting and delivery efficiency.^13^^,^^14^

### Ionizable lipids

Permanently charged cationic lipids were first used to formulate LNPs, which can bind electrostatically with the negatively charged phosphate backbones of nucleic acids. The strong electrostatic interactions facilitate the condensation of nucleic acids into stable nanoparticles. Cationic lipid 1,2-di-O-octadecenyl-3-trimethylammonium-propane (DOTMA) or 1,2-dioleoyl-3-trimethylammonium-propane (DOTAP) has been applied either alone or in combination with other materials for nucleic acid delivery.^15^^,^^16^^,^^17^^,^^18^^,^^19^^,^^20^^,^^21^^,^^22^^,^^23^^,^^24^ However, cationic lipids exhibit high toxicity as well as high immunogenicity.^25^ To address these issues, more advanced ionizable lipids have been developed. Ionizable lipids can be divided into three parts: (1) the ionizable polar head group, (2) the linker, and (3) two or more hydrophobic hydrocarbon tails.^26^ Ionizable lipids typically have a p*K*a value between 6.2 and 6.5, allowing them to be protonated and positively charged at a low pH environment, while remaining neutral at physiological pH.^27^ Notably, ionizable lipids largely determine the efficiency of encapsulation, endosome escape, and transfection.^5^^,^^28^^,^^29^^,^^30^^,^^31^

**Figure 2:**
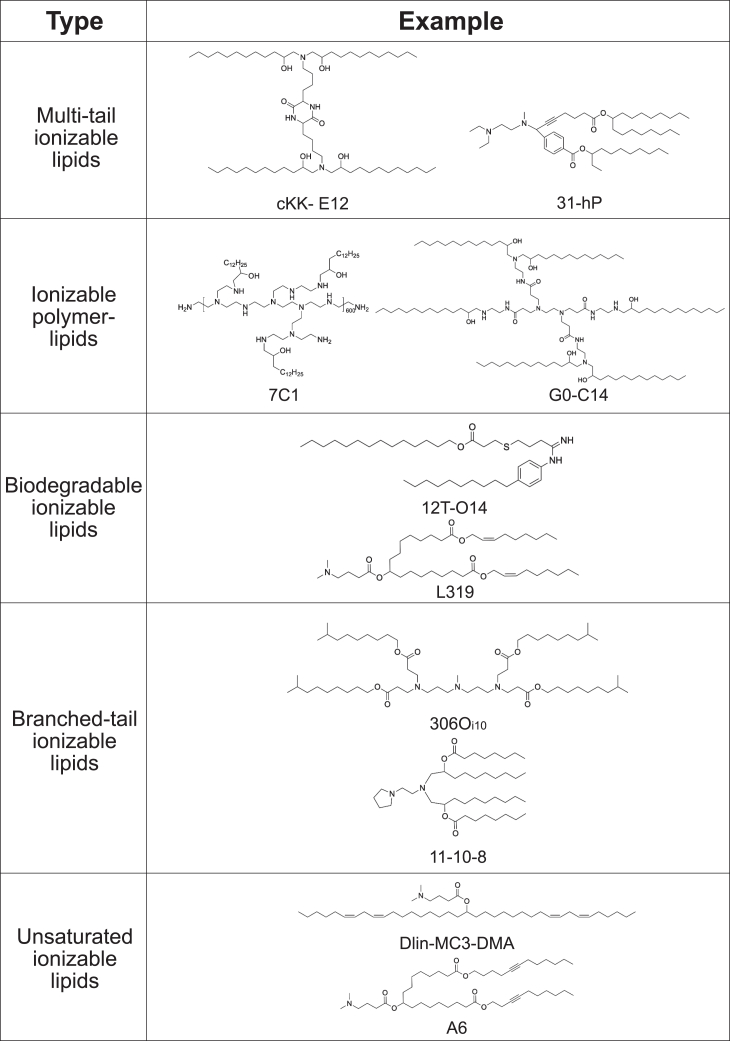
Classification and examples of ionizable lipids Ionizable lipids can be divided into five different categories: multi-tail ionizable lipids (e.g., cKK-E12^34^ and 31hP^35^), ionizable polymer-lipids (e.g., 7C1^36^ and G0-C14^37^), biodegradable ionizable lipids (e.g., 12T-O14^38^ and L319^39^), branched-tail ionizable lipids (e.g., 306Oi10^40^ and 11-10-8^9^), and unsaturated ionizable lipids (e.g., Dlin-MC3-DMA^27^ and A6^41^).

In recent years, many ionizable lipids have been developed and some have been approved by Food and Drug Administration (FDA). For example, DLin-MC3-DMA (MC3) was used in the first FDA-approved siRNA drug for the treatment of hereditary transthyretin amyloidosis (hATTR), which confers robust hepatic gene silencing^32^; SM-102 was applied in the COVID-19 mRNA vaccine mRNA-1273 approved by FDA in 2020.^33^ In addition to these FDA-approved benchmark ionizable lipids, more additional ionizable lipids have entered clinical trials or reported in preclinical studies. Due to the lack of categorization of ionizable lipids, Han et al. systematically classified them into five classes based on the structure (Figure 2), including multi-tail ionizable lipids, ionizable polymer-lipids, biodegradable ionizable lipids, branched-tail ionizable lipids, and unsaturated ionizable lipids.^42^

There are two main strategies for synthesizing ionizable lipids: medicinal chemistry and combinatorial chemistry. Medicinal chemistry is the original method used for the ionizable lipids synthesis,^43^ in which the head, linker, and hydrophobic tail are combined through multi-step standard chemical reactions. This synthetic strategy is labor-intensive and resource-consuming and has low throughput. In contrast, combinatorial chemistry enables fast, high-throughput, and combinatorial synthesis, which reduces cost, time, and resources. Common combinatorial chemistries include Michael addition, epoxide-mediated ring-opening reaction, reductive amination, and Ugi multicomponent reaction.^44^

Additionally, novel synthesis methods are being developed rapidly to produce new ionizable lipids. For example, Li et al. developed a biochemical synthesis method to enable one-step synthesis of biodegradable ionizable lipids.^45^ Recently, Han et al. utilized A^3^ (amine-aldehyde-alkyne) coupling reaction to synthesize propargylamine-based ionizable lipids and developed a directed chemical evolution approach to optimize their structures with improved delivery activity and biodegradability.^35^

Despite the synthetic advancement, identifying ionizable lipids with high potency and extrahepatic targeting ability remains time-consuming. In addition to traditional screening methods, machine learning is expected to accelerate the discovery of novel ionizable lipids with high potency and organ specificity. While some progress has been achieved in this area, more opportunities are available.^46^^,^^47^

### Phospholipids

Phospholipids, also known as helper lipids, are essential in LNP formulation. They can support the stability of LNPs by providing structural integrity and modulating the fluidity of the lipid bilayer.^23^ 1,2-distearoyl-snglycero-3-phosphocholine (DSPC), 1,2-dioleoyl-sn-glycero-3-phosphoethanolamine (DOPE), and 1,2-dioleoyl-sn-glycero-3-phosphocholine (DOPC) are frequently used.^48^ DSPC contains two saturated fatty acid chains with a rigid structure, which enables the formation of a lamellar phase and enhances the stability of LNPs.^49^ Notably, it is used in two marketed COVID-19 vaccines BNT162b2 and mRNA-1273.^50^ DOPE with two unsaturated fatty acid chains is known for its fusogenic properties, which tends to form an inverted hexagonal H(II) phase that can destabilize the endosomal membrane.^49^^,^^51^ Therefore, DOPE can facilitate the endosome escape of LNPs and improve delivery efficacy.^49^^,^^51^ DOPC contains a phosphocholine head and two unsaturated hydrocarbon tails. Qiu et al. showed that DOPC outperformed DSPC and DOPE in delivering mRNA-based gene editors.^52^

Phospholipids can also influence the biodistribution of LNPs *in vivo*. Zhang et al. found that DOPE promoted the hepatic accumulation of C12-200 LNPs, while DSPC favored splenic accumulation.^53^ Interestingly, although many studies have confirmed the critical role of phospholipids, Fei et al. found that phospholipids were not necessary in their simplified LNPs for tissue-targeted mRNA delivery.^54^

### Cholesterol

Cholesterol is a critical component of LNPs. It serves multiple functions, including enhancing particle stability, modulating membrane integrity, and regulating rigidity.^17^^,^^23^^,^^50^ Cholesterol can integrate into the lipid bilayer and improve the compactness of LNPs, thereby preventing the premature disassembly and increasing the nucleic acid delivery efficiency. The molar ratio of cholesterol in LNPs is typically 50%. The content of cholesterol can affect the formation and function of LNPs. Kawaguchi et al. observed when the percentage of cholesterol in LNPs was reduced from 40% to 10%, the cellular internalization and protein expression decreased significantly.^55^

Apart from cholesterol, researchers have explored many cholesterol derivatives. It was reported that replacing cholesterol with β-sitosterol or some oxidized cholesterol derivatives could improve the mRNA delivery efficiency.^56^ Patel et al. found that the use of β-sitosterol in enhanced LNP (eLNP) improved mRNA transfection ability.^57^ Compared with the conventional LNP, the cellular uptake and endosome escape were enhanced for eLNP. Interestingly, Liu et al. revealed that cholesterol was not crucial for LNP functionality.^58^ The removal of cholesterol could address the persistent challenge of liver accumulation and mediate extrahepatic targeting, demonstrating the promise of simplified LNPs for non-liver mRNA delivery.

Despite that different findings regarding cholesterol have been reported, cholesterol or its derivatives are generally considered as the important composition of LNPs, which can affect the stability, delivery efficiency, and targeting capability.

### PEG-lipids

PEG is a hydrophilic polymer, and when conjugated to lipids, it provides an anti-fouling PEG shell around the LNP, which increases the stability of LNPs, reduces the recognition and clearance by the immune system, and prolongs the circulation time *in vivo*.^59^ Commonly used PEG-lipids include 1,2-dimyristoyl-rac-glycero-3-methoxypolyethylene glycol-2000 (DMG-PEG2000) and 1,2-distearoyl-sn-glycero-3-phosphoethanolamine-N-[methoxy (polyethylene glycol-2000)] (DSPE-PEG2000).

PEG-lipids can affect the physicochemical properties and functionalities of LNPs, such as polydispersity, size, encapsulation efficiency, transfection efficiency, and immune responses. For example, LNP containing DMG-PEG2000 showed better delivery capability than DSG-PEG2000 or DSPE-PEG 2000.^60^ The length of the acyl chain critically affects the transfection potency of LNPs. The PEG-lipid with two shorter acyl chains generally enhances the cellular uptake and gene delivery due to its rapid disassociation from LNPs.^61^

A major concern in using PEG-lipids is their immunogenicity. Some studies have shown that repeated administration of PEGylated LNPs can induce the production of anti-PEG antibodies, which accelerate the clearance of subsequent doses and reduce therapeutic efficacy, known as the “accelerated blood clearance” (ABC) effect.^62^ The anti-PEG antibodies also relate to the allergic response or other adverse reactions. Some efforts have been made to optimize PEG structure or develop alternative anti-fouling polymers with reduced immunogenicity, including polyoxazoline, polyvinyl alcohol, and polyglycerol.^63^^,^^64^^,^^65^^,^^66^^,^^67^^,^^68^ Recently, Kang et al. showed that a polysarcosine (pSar) lipid increased or maintained mRNA delivery efficiency and exhibited similar safety profiles *in vivo* compared to the PEG-lipid.^69^ More investigations are needed to verify the safety and effectiveness of these PEG-lipid alternatives before their clinical use.

## Formulation techniques

**Figure 3:**
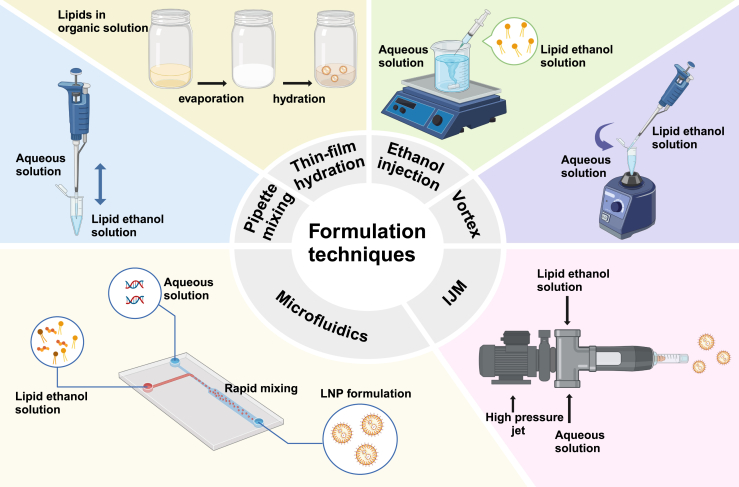
LNP formulation techniques The schematic illustration of main LNP formulation techniques, including pipette mixing, vortex, thin-film hydration, ethanol injection, microfluidics, and impingement jet mixing (IJM), is shown.

The LNP formulation technique plays an important role in the physicochemical properties, encapsulation efficiency and stability, which directly affect the quality, storage, and performance of LNPs.^70^^,^^71^ In this section, we discuss the main LNP formulation techniques used in the laboratory and industry, including pipette mixing, vortex, thin-film hydration, ethanol injection, microfluidics, and impingement jet mixing (IJM) (Figure 3).

### Pipette mixing

Pipette mixing is a rapid formulation technique, which is suitable to prepare a small batch of LNPs. The organic solution containing lipids and the buffer solution containing nucleic acids are combined and quickly mixed by pipetting.^72^ The advantages of this method include simplicity and time efficiency. Moreover, high-throughput LNP formulation can be achieved with a multi-channel pipette. Chen et al. used pipette mixing to prepare small batches of iGeoCas9 RNP-LNPs for genome-editing applications.^73^ Xu et al. efficiently prepared LNPs by the pipette mixing method to enable their AI-guided ionizable lipid engineering platform.^46^ However, this technique is limited by poor reproducibility and low scalability.^74^ Moreover, the formulated LNPs are poly-dispersed and colloidally unstable with low nucleic acid encapsulation efficiency.

### Vortex

Vortex mixing can quickly prepare small-scale batches of LNPs with reduced labor input compared to pipette mixing.^72^ In this method, the buffer solution is combined with lipid ethanol solution and then vortexed at a moderate speed on the vortex mixer.^72^ It is a conventional method for LNP production, and is easy to implement and operate at a laboratory scale. Vortex is generally used for bench-scale volumes (100 μL–4 mL).^72^ The limitations of this mixing technique are similar to pipette mixing discussed previously.^72^^,^^75^^,^^76^

### Thin-film hydration

Thin-film hydration is a classic technique to prepare LNPs.^77^ Lipids are first dissolved in an organic solvent, such as ethanol and chloroform, which is removed by evaporation to form a lipid film. Then, the lipid film is hydrated by aqueous media containing nucleic acids to form a sealed spherical structure.^78^ Finally, LNPs are homogenized by extrusion or ultrasonication.^79^ Thin-film hydration can generate bench-scale LNPs with minimal residual solvent, simplified production procedures, and low cost. However, when the production quantity increases, the volume of organic solvent increases. Therefore, it could take several hours to evaporate the organic solvent, which is time-consuming.^78^ Besides, this method also has limitations of low encapsulation efficiency and scale-up challenges.^80^

### Ethanol injection

Ethanol injection is a general technique to formulate LNPs. It was first introduced and developed in 1973 by Batzri and Korn.^81^ The lipids are dissolved in ethanol and quickly injected into the aqueous buffer containing nucleic acids for encapsulation while stirring. The injected lipid solution is rapidly diluted by aqueous buffer, thus forming vesicles due to the increase of polarity in the mixed solvent.^82^ The size of LNPs formed is influenced by lipid concentration, stirring rate, injection rate, and the lipids used.^82^^,^^83^ Zhang et al. formulated a one-component multifunctional ionizable amphiphilic Janus dendrimer LNP using this method.^84^ However, the drawbacks are the lack of reproducibility, scalability, and low encapsulation efficiency, which hinder its applications in many cases.^85^ While traditional ethanol injection is used to produce bench-scale LNPs, cross-flow injection is developed for mass production. In this case, the ethanol solution is injected into the buffer solution in a device with a cross-flow tube, which can control the flux by changing the pressure of the nitrogen-regulating device.^86^

### Microfluidics

Microfluidic technology has been widely used in the fields of chemical synthesis, diagnosis, crystallization, nanoparticle synthesis, and high-throughput screening.^87^^,^^88^ It has become a powerful and reliable technique to formulate LNPs.^74^ Compared with traditional methods, the microfluidics enable more uniform and reproducible nanoparticles at the microscale.^89^ Developments in structured microfluidic chips have perfected the mixing process, leading to homogeneous particle size and high encapsulation.^90^^,^^91^ The microfluidic approach allows for precise tuning of formulation parameters, such as flow rates, concentrations, and mixing ratios. By controlling these parameters, researchers can optimize the size, encapsulation capacity, and stability of LNPs.^92^ In a microfluidic device, LNPs are produced by the ethanol-dilution method generally, where the lipid ethanol solution is rapidly mixed with the aqueous phase containing nucleic acids, leading to the self-assembled complexes due to the electrostatic interactions.^93^

### Microfluidic hydrodynamic flow focusing

Microfluidic hydrodynamic flow focusing (HFF) is one of the mostly used micromixer designs. This microfluidic laminar flow method can facilitate rapid mixing between the two fluids, in which a narrow fluid stream flows in the same channel next to the other.^74^ By adjusting the flow rate ratio (FRR) or the total flow rate, uniformly dispersed LNPs with different sizes can be produced.^94^

Traditional HFF devices have a 2D structure belonging to chip-based platforms.^85^ The ethanol phase containing the precursor ingredients of the LNPs is introduced to the central, then generating rapid diffusion-based mixing. However, the low flux of 2D HFF affects the particle size control, leading to channel blockage.^95^ An advanced 3D HFF device was developed based on capillary platforms, in which the central flow of ethanol is focused radially by aqueous buffer.^85^ Compared to 2D HFF devices, the throughput was increased by 4-folds without affecting the size of LNPs.^96^

HFF does not require time-consuming treatments, such as ultrasonication, extrusion, and filtration,^94^^,^^96^ making it suitable to produce LNPs in industry.^94^ However, the main disadvantages of HFF are high cost and complicated manufacturing operations. Besides, the high FRR dilutes the sample, which increases the workload for subsequent concentration.^12^

### Microfluidic staggered herringbone micromixers

Cullis and his coworkers pioneered the use of microfluidic staggered herringbone micromixers (SHMs) to produce LNPs.^97^ Due to the success of SHM devices, Precision NanoSystems commercialized this architecture for nanoparticle production.^98^ SHMs contain an asymmetric herringbone groove pattern, which disrupts the laminar flow, leading to chaotic advection and controlled mixing (<10 ms) to form homogeneous LNPs with high reproducibility.^74^^,^^99^ The structure of staggered herringbone mixers is beneficial to effectively mix two fluids, so that the interface between the fluids expands exponentially, ensuring rapid formulation.^100^

However, this method has low flux, which becomes a major bottleneck to large-scale LNP production.^101^ To address this issue, a revolutionary system named parallel microfluidic device based on SHMs was developed. It provides an impressive 100-fold enhancement of flux on a single microfluidic channel, and meets the urgent demand for efficient and multifunctional manufacturing process in LNP production.^70^ Belliveau et al. used SHMs to enable the routine production of siRNA-LNP in the size range 20–100 nm, with equivalent or better gene silencing potency compared to the previous formulation technique.^90^ In another study, Shepherd et al. presented a silicon scalable lipid nanoparticle generation platform with a branching architecture. This platform incorporated 256 SHM mixing, which can be applied to scalable LNP formulation and accelerate the production of LNP-based RNA therapeutics and vaccines.^102^ SHM, as a very effective method, can greatly promote the clinical development and application of LNPs.

### T-junction mixing

In 1999, Hirota et al. introduced T-junction mixing to produce a lipid-based drug, providing an alternative to macroscopic mixing methods.^103^ T-junction mixing is a rapid mixing method requiring high flow rates (40–60 mL/min) to produce large volumes of LNPs.^74^ The two input flows collide in a T-junction, leading to speedy mixing and a turbulent output.^104^

Compared with macro-mixing methods, T-junction mixing produces repeatable and controllable LNPs.^105^ For companies engaged in siRNA-LNPs, T-junction mixing is a preferred method for mass production.^106^^,^^107^ However, it needs strict regulation to ensure the high flow rate and rapid mixing.^108^ For example, Abrams et al. combined a T-type microfluidic device and a high-performance liquid chromatography pump with a solution-flow rate of 40 mL/min to formulate siRNA-loaded LNPs.^93^ Overall, T-junction mixing offers an alternative method for large-scale LNP production.

### Impingement jet mixer

IJM is an innovative microfluidic mixing technology using high-speed fluid flow for LNP production.^109^ In this process, IJM is used to mix the lipid phase with the aqueous solution, forming small droplets through a high-pressure jet and finally being stabilized by surfactants.^110^ By adjusting the parameters of the mixer, such as pressure and flow rate, the physicochemical properties of nanoparticles can be controlled. During COVID-19, IJM systems were widely used for mRNA vaccine production by several companies, including Pfizer.^70^ Knauer also encapsulated the antigen-encoding mRNA into LNPs using IJM, which ensured the bioavailability and effectiveness of mRNA.^111^

## Application

**Figure 4:**
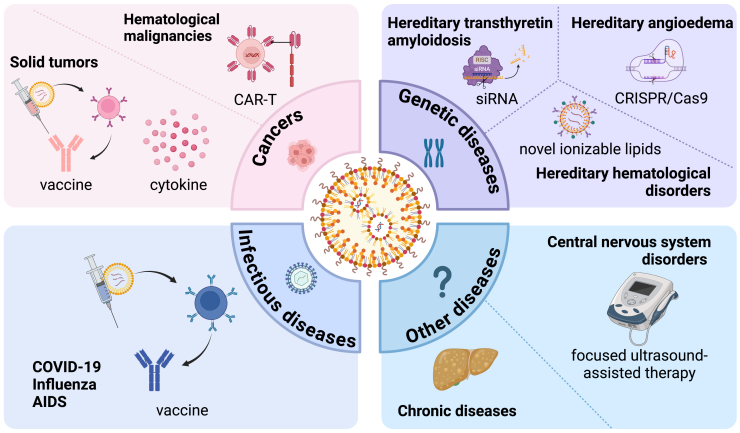
The applications of LNPs in various diseases Nucleic-acid-loaded LNPs show great promise in the prevention or treatment of cancers, infectious diseases, genetic diseases, and many other diseases.

As non-viral vectors to encapsulate and deliver nucleic acids to target tissues and cells, LNPs demonstrate substantial advantages and potential in the development of nucleic acid drugs, especially RNA drugs. In this section, we discuss the applications of nucleic-acid-loaded LNPs in the prevention or treatment of various diseases, including cancers, infectious diseases, genetic disorders, and so on (Figure 4).

### Cancers

Cancer is one of the major public health problems, which causes approximately a quarter of deaths from noncommunicable diseases worldwide.^112^ LNP-mediated therapies are widely applied in hematological malignancies and solid tumors by delivering nucleic acid drugs in preclinical and clinical studies.

### Hematological malignancies

Hematological malignancies include leukemia, lymphoma, and multiple myeloma.^113^ Adoptive cell therapy has demonstrated great efficacy in the treatment of hematological malignancies.^114^ Nowadays, seven chimeric antigen receptor (CAR)-T cell products produced by virus vectors have received FDA approval. However, the permanent CAR expression poses the risk of severe adverse effects, such as cytokine release syndrome.^115^ Moreover, these CAR-T cells could potentially trigger the secondary cancer due to the insertional mutagenesis.^116^

To address these issues, LNPs have been used to generate CAR-T cells by delivering CAR mRNA, which could revolutionize the adoptive cell therapy.^67^ For *ex vivo* CAR-T therapy, T cells from peripheral blood are engineered to express CAR, which are further infused back to patients/donors.^117^ For instance, Billingsley et al. used the optimized C14-4 LNP formulation to produce anti-CD19 CAR-T cells *ex vivo*, which elicited strong cancer-killing activity at levels equivalent to electroporation.^66^^,^^118^ In another study, Ye et al. developed 76-O17Se LNP, which could efficiently deliver CAR mRNA into T cells, leading to the robust CAR-T cell cytotoxicity.^119^ Although *ex vivo* CAR-T therapy is feasible, the high manufacturing cost and long manufacturing cycle largely restrict its broad application.^117^

*In vivo* CAR-T therapy enabled by LNPs is also under investigation. However, this strategy requires a high degree of cell- or organ-targeting specificity.^120^ Álvarez-Benedicto et al. leveraged spleen selective organ targeted LNPs by using 18:1 PA as a fifth component to generate CAR-T cells *in situ*, which increased the overall survival of B cell lymphoma-bearing mice.^121^ Besides, Billingsley et al. modified LNPs with different T cell-targeting antibodies (Ab-LNPs) to enhance spleen delivery, and found that CD3-LNPs and CD7-LNPs worked better than CD5-LNPs to generate functional anti-CD19 CAR-T cells with B cell depletion rate up to 90%.^122^ Recently, Capstan reported that their *in vivo* anti-CD19 CAR-T cell therapy enabled by CD5- or CD8-tLNPs could clear Nalm6 tumor with high efficiency and specificity in a humanized mouse model, highlighting the potential for clinical development.^123^

### Solid tumors

LNP-based therapies have also been applied to treat solid tumors, such as mRNA vaccines, cytokine-encoded mRNA therapeutics, gene silencing, and gene editing. mRNA vaccines are considered as an effective method to treat solid tumors, with a focus of personalized cancer vaccines. LNPs can deliver antigen-encoded mRNA into antigen-presenting cells, which activate CD4^+^ T cells and CD8^+^ T cells to kill antigen-expressing tumor cells.^124^ To design efficient mRNA vaccines, many efforts have been put into optimizing both the mRNA cargoes and LNPs. BioNTech selected multiple mutation-derived neoantigens from pancreatic ductal adenocarcinoma patients, and used their LNPs to deliver neoantigen-encoded mRNAs intravenously into patients. This personalized vaccine named autogene cevumeran (BNT122) has shown promising therapeutic outcomes.^125^ Besides, the potency of mRNA vaccines can be enhanced by refining the structure and components of LNPs to achieve high delivery efficiency and organ specificity. Chen et al. developed an endogenous lymph node-targeting 113-O12B LNP to deliver OVA-encoding mRNA, which elicited a strong CD8^+^ T cell response with excellent protective and therapeutic effects against B16F10 melanoma.^126^ Nowadays, several mRNA-LNP cancer vaccines are being tested in clinical trials, underscoring the great promise of LNPs in tumor prevention and therapy (Table 1).

**Table 1:** Representative mRNA-LNP cancer vaccines in clinical trials

Name	Encoded antigen	Administration route	Condition	Stage	NCT number
BNT112	kallikrein-2, kallikrein-3, acid phosphatase prostate, homeobox B13, and NK3 homeobox 1	i.v.	prostate cancer	phase 1/2 study	NCT04382898^127^
BNT113	HPV-16 oncoproteins E6 and E7	i.v.	head and neck cancer	phase 2 study	NCT04534205^128^
BNT122	up to 20 neoantigens	i.v.	pancreatic cancer	phase 1 study	NCT04161755^125^
mRNA-4157	up to 34 neoantigens	i.m.	non-small cell lung cancer	phase 3 study	NCT06077760^129^
	up to 34 neoantigens	i.m.	melanoma	phase 3 study	NCT05933577^130^
mRNA-5671	4 prevalent *KRAS* mutant antigens	i.m.	tumors with *KRAS* mutation	phase 1 study	NCT03948763^131^

In addition to the mRNA vaccines, certain cytokines can inhibit tumor initiation and progression, and are being tested in cancer therapy. Interleukin (IL)-12 is a well-known candidate to regulate T lymphocytes and natural killer cell responses, leading to the production of interferon (IFN)-γ to kill tumor cells.^132^ Hewitt et al. designed IL-12/mRNA-LNP, which were intratumorally injected to promote the TH1 tumor microenvironment transformation, resulting in robust antitumor immunity.^133^ This research has progressed to the clinical stage (MEDI1191).^134^

Besides, the immune checkpoint can be silenced alone or in combination with other therapeutic modalities to combat tumors. For example, a novel nanovaccine (dClip-LNP/siRNA) was designed to activate Toll-like receptors and silence T cell immunoglobulin and mucin-domain containing-3 (TIM3) simultaneously.^135^ Recently, Zhang et al. used LNPs to co-deliver siRNA, Cas9 mRNA, and single-guide RNA (sgRNA) into tumors to knock down focal adhesion kinase and knock out PD-L1, which significantly inhibited tumor growth and metastasis in four mouse models of cancer. This study offers a paradigm for synergistic therapy.^136^

### Infectious diseases

Over the past few decades, public health has been threatened by various infectious diseases.^137^ Due to the scope, we mainly discuss the applications of LNPs in the fight against two respiratory diseases (COVID-19 and influenza) and acquired immuno-deficiency syndrome (AIDS) (Table 2).

**Table 2:** Representative mRNA-LNP clinical development for infectious diseases

Drug name	Administration route	Target virus	Stage
mRNA-1273	i.m.	COVID-19	approved
BNT162b2	i.m.		approved
ARCT-154	i.m.		approved
DCVC H1 HA mRNA vaccine	i.m.	H1N1	phase 1 study
H3 mRNA/LNP vaccine	i.m.	H3N2	phase 1 study
mRNA-1769	i.m.	MPXV	phase 1/2 study

### COVID-19

As an innovative approach to combating infectious diseases, mRNA vaccines have gained great attention in COVID-19 prevention. Two COVID-19 mRNA-LNP vaccines (mRNA-1273 and BNT162b2) were approved by FDA for emergency use within a year of pipeline development.^138^ The published clinical data showed that these vaccines resulted in strong immune responses and high levels of spike-specific neutralizing antibodies in vaccine recipients.^6^^,^^139^^,^^140^ LNPs were also used in the first self-amplifying RNA vaccine approved in Japan to combat COVID-19, which used a lower dose compared to mRNA-1273 and BNT162b2.^141^

### Influenza

Influenza A and B virus infections cause annual seasonal epidemics, leading to a significant global disease burden.^142^ Considering the conserved antigenic epitopes in various influenza viruses, it is an effective strategy to design multivalent vaccines. mRNA-LNPs can facilitate the development of universal influenza vaccines. Arevalo et al. designed a nucleoside-modified mRNA-LNP vaccine encoded hemagglutinin from all 20 known influenza A virus subtypes and influenza B virus lineages,^143^ which could produce multiple antibodies and protect mice from the virus. To combat H1N1 and H3N2 influenza viruses, mRNA-LNP vaccines (NCT05945485 and NCT05829356) are undergoing clinical trials to evaluate safety and immunogenicity.^144^^,^^145^

### AIDS

Although HIV-related research has lasted over 40 years, AIDS is still a significant global health challenge, affecting 38 million people.^146^ The development of vaccines for AIDS has progressed slowly due to several bottlenecks, including the genetic diversity of HIV and its immune evasion.^147^ The flexibility and strong immunogenicity of mRNA vaccines hold the promise to prevent HIV infection. Xie et al. developed an mRNA-LNP vaccine, which was effective against HIV and generated long-lasting germinal centers to enhance the immunogenicity.^148^ Apart from vaccines, Pardi et al. developed a viable platform for passive immunotherapy against HIV, where LNPs were loaded with VRC01 mRNA encoding the light and heavy chains of neutralizing anti-HIV-1 antibody.^149^ Nevertheless, more efforts are needed in both preclinical and clinical research to combat AIDS using mRNA-LNP technology.

### Other infectious diseases

LNPs have also been used to fight against other infectious diseases. For example, since the outbreak of Zika virus in 2015, several mRNA-LNP vaccines have been developed.^150^ Pardi et al. developed an mRNA-LNP vaccine encoding the pre-membrane and envelope glycoproteins, which induced potent and durable neutralizing antibody responses in mice and non-human primates.^151^

Besides, mRNA-LNP vaccines have demonstrated significant advantages in the prevention of mpox virus. mRNA-1769 is a mRNA-LNP vaccine developed by Moderna, which demonstrates enhanced viral control and disease attenuation.^152^ This vaccine has progressed to phase 1/2 clinical study (NCT05995275).^153^

### Genetic diseases

Genetic diseases have complex and challenging pathological characteristics due to the changes in the genetic code.^154^ There are many genetic diseases; here we mainly discuss the applications of LNPs in the treatment of hereditary liver and hematological diseases. It is worth mentioning that LNP-enabled gene-editing therapies are undergoing clinical development (Table 3), which hold the potential to cure genetic diseases permanently in a single treatment.

**Table 3:** Representative mRNA-LNP clinical development for gene editing

Name	Gene-editing technology	Administration route	Condition	Stage
NTLA-2001	CRISPR-Cas9	i.v.	hATTR	phase 3 study
NTLA-2002	CRISPR-Cas9	i.v.	HAE	phase 3 study
VERVE-101	base editing	i.v.	HeFH	phase 1 study

HeFH, heterozygous familial hypercholesterolemia.

### hATTR

Transthyretin (TTR) amyloidosis occurs due to the accumulation of TTR amyloid, which is produced by hepatocytes.^155^ Over 100 genetic variants of the *TTR* gene are linked to autosomal dominant familial amyloidotic polyneuropathy. Disruption of the expression of pathological TTR holds great potential for the treatment of hATTR.^156^

Onpattro (Patisiran) is the first approved siRNA drug for the treatment of hATTR.^157^ It ensures the robust inhibition of mutant TTR protein production and the subsequent fibril formation by efficiently delivering TTR siRNA into hepatocytes using MC3 LNPs.^32^^,^^157^ Due to the live tropism of MC3 LNP, Patisiran demonstrated superior clinical benefits in hATTR with polyneuropathy, and its safety has been well proved.^55^

In addition to the RNA interference strategy, Clustered Regularly Interspaced Short Palindromic Repeats (CRISPR) technology is also applied in the treatment of hATTR. Gillmore et al. found LNPs encapsulating Cas9 mRNA and an sgRNA targeting TTR (NTLA-2001) could effectively reduce the level of pathogenic protein by at least 52% in the peripheral blood of patients, thereby alleviating disease progression.^158^ NTLA-2001 is now in phase 3 clinical trial (NCT06672237 and NCT06128629).^159^^,^^160^

### Hereditary angioedema

Hereditary angioedema (HAE) is a rare genetic disorder manifested by cutaneous and submucosal swelling.^161^ The absent inhibition of the enzyme kallikrein leads to the over-production of bradykinin, which is identified as the mediator of swelling in HAE.^162^ NTLA-2002 is a gene-editing therapy combining LNPs with mRNA-based gene editors to target and knock out the *KLKB1* gene in the liver, which permanently inhibits the production of kallikrein and subsequently reduces the generation of bradykinin.^163^ In phase 1 or 2 clinical trial, a single dose of NTLA-2002 led to the robust decrease of kallikrein level with good safety and tolerability profiles observed across all doses (NCT05120830).^164^

### Hereditary hematological disorders

Hereditary hematological disorders are a group of blood system diseases caused by genetic mutations, such as thalassemia and sickle cell disease (SCD).^165^ Thalassemia is a genetically diverse group of disorders that impacts globin chain synthesis, which is distributed globally.^166^ According to the mutation types, thalassemia can be classified into α-thalassemia and β-thalassemia.^167^ Hematopoietic stem cells (HSCs) are general targets for gene therapy in hematological disorders and have been widely employed in many studies. An antibody-free targeted LNP was developed by Xu et al., which enabled efficient base editing in HSCs, leading to the restored globin chain balance in erythroid cells.^168^

SCD is caused by the substitution of glutamine-to-valine, resulting in sickle hemoglobin and obstructing blood circulation.^169^ Breda et al. developed an anti-human CD117/LNP-based base-editing system that targeted HSCs. Loaded with mRNA encoding a Cas9 adenine base editor and an sgRNA targeting the β-globin sickle cell mutation, the CD117/LNP led to near-complete correction of hematopoietic sickle cells *ex vivo*.^170^

### Other diseases

Apart from the aforementioned scenarios, LNPs have also been applied in other pathological situations, such as central nervous system disorders. To efficiently deliver therapeutics to treat neurological diseases, LNPs must cross the blood-brain barrier (BBB) and achieve specific targeting within the brain parenchyma.^171^ Microbubble-assisted focused ultrasound (FUS) is utilized to temporarily increase BBB permeability. For example, Ogawa et al. demonstrated that targeted delivery of ZsGreen1-coding mRNA to microglial cells and endothelial cells can be achieved by LNPs with the assistance of FUS technology.^172^ Wu et al. designed a borneol-modified LNP to increase BBB permeability, which could deliver exenatide to reduce α-synuclein expression and Lewy bodies deposition of PD mice.^173^

Besides, LNPs are used for the treatment of chronic diseases, such as hepatic steatosis. For example, vascular endothelial growth factor A mRNA was delivered by LNPs to accelerate biliary epithelial cell (BEC)-to-hepatocyte conversion, reverse steatosis and fibrosis.^174^ Recently, Han et al. devised a novel construction strategy to synthesize degradable branched lipidoids and demonstrated that repeated administration of fibroblast growth factor 21 mRNA-loaded DB-LNPs could ameliorate hepatic steatosis in obese mice.^175^

## Conclusion

LNPs have demonstrated enormous potential in nucleic acid therapeutics. Altering the composition and ratio of traditional formulations can be used to optimize LNPs. Moreover, the addition of new components, such as charge-adjusting lipids and targeting ligands, can dramatically change the tropism and targeting ability of LNPs.^176^^,^^177^^,^^178^ Therefore, optimizing the composition and formulation is crucial for the biomedical applications of LNPs.

For LNP formulation techniques, researchers often use pipette mixing or vortex to prepare small-scale LNPs in the laboratory. These methods are simple and convenient, but the prepared LNPs are inhomogeneous and unstable with low encapsulation efficiency. Classic methods (e.g., ethanol injection and thin-film hydration) are economic but are limited by low encapsulation efficiency and poor repeatability. The microfluidic technique is now widely used to formulate homogeneous LNPs in both laboratory and industry, yet the high cost cannot be overlooked. Researchers should choose the appropriate LNP formulation approach based on specific scenarios.

LNPs are the lead non-viral vector for the delivery of nucleic acids, including mRNA, siRNA, and CRISPR components. With the success in siRNA-LNP drugs and mRNA-LNP vaccines, the next breakthrough could be LNP-based gene-editing therapy for genetic diseases. Besides, LNPs are also popular in personalized cancer mRNA vaccines and *in vivo* CAR-T therapy, both of which could revolutionize the cancer therapy. With the rise of nucleic acid drugs, especially RNA drugs, the development of LNP technology will be increasingly crucial in the prevention or treatment of various diseases.

In the future, artificial intelligence will play an increasing role of next-generation LNP development by accelerating LNP design and screening. Besides, high-throughput sequencing technologies (e.g., RNA sequencing) will facilitate tumor neoantigen identification and individualized mRNA vaccine design. Therefore, LNP-enabled RNA drugs represent a transformative approach in precision and personalized medicine.

## Acknowledgments

X.H. acknowledges the support from the Strategic Priority Research Program of the Chinese Academy of Sciences (XDB0570000), the 10.13039/501100001809National Natural Science Foundation of China (32471401), and an independent project of Shanghai Sci-Tech Inno Center for Infection & Immunity (ssIII-2024B01). Y.X. acknowledges the support from the Shanghai Pujiang Program of Magnolia Talent Plan (24PJA028).

## Author contributions

S.X., Z.H., and F.S. searched the data, wrote the article, and created figures. Y.X. and X.H. conceived, wrote, and edited the article.

## Declaration of interests

The authors declare no competing interests.
